# Sorafenib pretreatment enhances radiotherapy through targeting MEK/ERK/NF-κB pathway in human hepatocellular carcinoma-bearing mouse model

**DOI:** 10.18632/oncotarget.13398

**Published:** 2016-11-16

**Authors:** John Chun-Hao Chen, Hui-Yen Chuang, Fei-Ting Hsu, Yi-Chen Chen, Yi-Chun Chien, Jeng-Jong Hwang

**Affiliations:** ^1^ Department of Biomedical Imaging and Radiological Sciences, National Yang-Ming University, Taipei, Taiwan; ^2^ Department of Radiation Oncology, Mackay Memorial Hospital, New Taipei City, Taiwan; ^3^ Department of Radiology, National Taiwan University Hospital, Taipei, Taiwan; ^4^ Department of Medical Imaging and Radiological Sciences, I-Shou University, Jiaosu Village, Kaohsiung, Taiwan; ^5^ Biophotonics and Molecular Imaging Research Center, National Yang-Ming University, Taipei, Taiwan

**Keywords:** hepatocellular carcinoma, sorafenib, radiation, MEK/ERK/NF-κB signaling, molecular imaging

## Abstract

Patients with unresectable hepatocellular carcinoma (HCC) usually have poor prognosis because current monotherapy including surgery, chemotherapy and radiotherapy (RT) are not effective. Combination therapy may be effective to overcome this clinical problem. Here, we proposed the combination of sorafenib and RT, which have been applied in HCC treatment, could improve the treatment outcome of HCC. Our previous study showed that sorafenib could suppress the expression of NF-κB which is related to the chemo- and radio-resistance. Nevertheless, the expression of NF-κB is oscillatory and is affected by the treatments. Thus, understanding the oscillation of NF-κB expression would be beneficial for determining the optimal treatment schedule in combination therapy. Here established Huh7/NF-κB-*tk-luc2/rfp* cell line, in which NF-κB indicates a NF-κB promoter, was utilized to noninvasively monitor the expression of NF-κB overtime *in vitro* and *in vivo*. The results show that pretreatment of sorafenib with RT suppresses the expressions of NF-κB and its downstream proteins induced by radiation through downregulation of phosphorylated extracellular signal-regulated kinase (pERK) most significantly compared with other treatment schedules. The results were further verified with Western blotting, EMSA, and NF-κB molecular imaging. These findings suggest that pretreatment of sorafenib with RT may be the ideal treatment schedule for the treatment of HCC.

## INTRODUCTION

Hepatocellular carcinoma (HCC) is the fifth common malignancy worldwide and the mortality is ranked the third in cancer-related deaths. Moreover, the incidence rate of HCC is continuously increasing [1, 2], particularly in the East Asia where the rate exceeds 30 cases per 100,000 people per year [3]. Though surgery is the best treatment for HCC, only a quarter of patients with HCC qualify for this modality. The prognosis of patients with unresectable HCC is poor, and chemotherapy and radiotherapy (RT) could benefit little to these patients [4]. Various combinations of chemotherapy, targeted therapy, and RT have been investigated to achieve the better treatment outcome [5–7]. However, mechanisms underlying hepatic carcinogenesis have not been fully unraveled. Neither suitable targets nor optimized treatment regimens for different stages of the disease yet identified, which are important for combining different therapies for HCC treatment.

Radiotherapy and chemotherapy usually are given separately to patients with unresectable HCC. Nevertheless, radio- and chemo-resistance are often developed after several treatment courses and result in unfavorable outcomes [8, 9]. Sorafenib, a multikinase inhibitor approved by U.S. Food and Drug Administration in 2009, has been shown to be a targeted drug for advanced HCC through the suppression of tumor growth and angiogenesis. The average survival time, however, only is extended for 2-3 months after receiving sorafenib treatment as compared with the control group [10].

The outcomes of most HCC cases treated with RT are not satisfied since the tolerance dose of normal liver is much lower than the dose required for the cure of the hepatoma. Leakage radiation dose delivered to the normal liver tissues surrounding tumor will result in radiation-induced liver disease (RILD) which appears when the mean liver dose is higher than 30 Gy [11]. However, RT is reconsidered to be recruited into the standard treatment of HCC after the development of stereotactic body radiotherapy (SBRT) [12, 13]. The dose delivered to the normal liver could be reduced with SBRT, thus lower the incidence of RILD. In addition, the charged particle therapy is also proposed for better control rate of HCC and could decrease the prevalence of RILD.

The therapeutic outcome of HCC with transarterial chemoembolization of RT could be improved when combined with sorafenib [14–16]. Sorafenib has been shown to sensitize both human colorectal and oral carcinomas to RT in tumor-bearing mouse models *via* the inhibition of nuclear factor kappa-light-chain-enhancer of activated B cells (NF-κB) and its downstream effector proteins [17, 18]. Thus, better outcome from sorafenib combined with RT seems to be achievable in the clinical setting for HCC treatment. Radiosensitivity of HCC cells enhanced by sorafenib using sequential regimens *in vitro* has been demonstrated but with controversial results, such that further validations are needed [19–21]. The low survival rate of monotherapy and the treatment resistance found in HCC might be overcome if combination therapy is adopted. Recently, clinical trials of combination of sorafenib and radiotherapy in HCC have been conducted, and better treatment results with high toxicity were found [22–24]. Treatment resistance found in HCC has been shown to be related to NF-κB activation [25, 26], which is involved in tumor invasiveness, metastasis, proliferation and antiapoptosis, all contribute to the malignancy of cancer cells [27, 28].

The unsatisfied outcome of HCC is most correlated with the oscillatory expression of NF-κB after chemotherapy or RT. Activation and dynamic oscillation of NF-κB are mainly controlled by IκB-regulated negative feedback [29, 30], and oscillation in transcription factor dynamics is an alternative way to control gene expressions [31]. For instance, extracellular signal-regulated kinase (ERK) could regulate the activation of NF-κB and its downstream genes temporally [32]. Simultaneous imaging of NF-κB activity and viable hepatoma cells reflects the real status and temporal change of NF-κB activity in tumor lesions after sorafenib treatment [33]. Here theHuh7/NF-κB-*tk-luc2*/*rfp* cell line was used to monitor the oscillation of NF-κB activity in HCC to determine the optimal schedule for sorafenib combined with RT through dynamic monitoring of NF-κB *in vivo* with molecular imaging. The underlying mechanism of radiosensitization of sorafenib was also investigated with different assays

## RESULTS

### Radiation enhances NF-κB activity in Huh 7/NF-κB-tk-luc2/rfp cells

**Figure 1:**
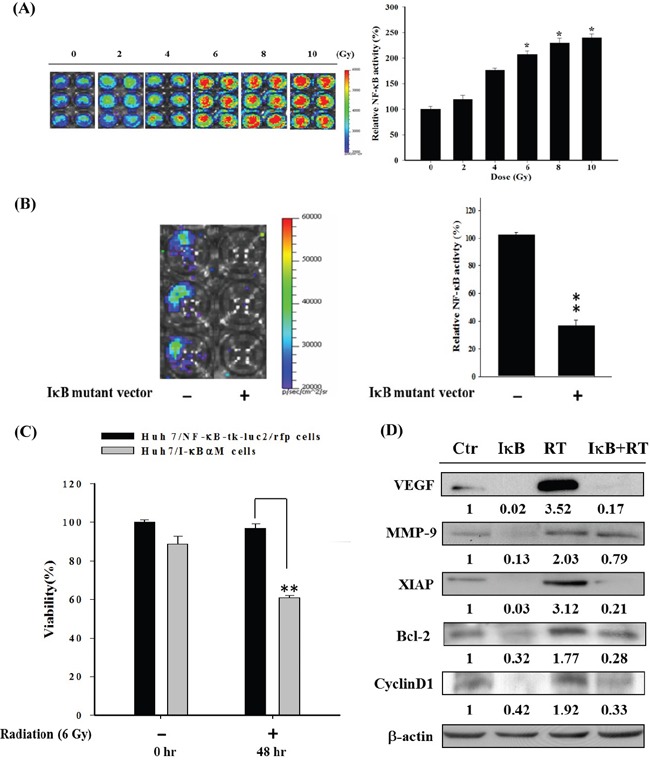
Radiation induces NF-κB activity in Huh7/NF-κB-*tk-luc2/rfp* cells **A.** Huh7/NF-κB-*tk-luc2/rfp* cells were irradiated with 0–10 Gy and the BLI was performed to assess the NF-κB activity assay 48 h postirradiation. Radiation enhanced NF-κB activity in a dose-dependent manner. Significant elevation of NF-κB activity was found when cells received irradiation higher than 6 Gy. **B.** The IκBαM vector was used as a positive control for NF-κB inhibition and was transfected into the Huh7/NF-κB-*tk-luc2/rfp* cells. The quantified results showed that endogenous NF-κB activity was strikingly repressed by transfection of IκBαM vector. **C.** Higher cytotoxicity caused by radiation was observed in the cells transfected with the IκBαM vector as compared with nontransfected cells. **D.** Radiation induced the expressions of NF-κB downstream proteins were suppressed by transfected with the IκBαM vector. (*p< 0.05, **p< 0.01, as compared with that of the control).

Figure 1A shows that the NF-κB activity was enhanced by radiation in a dose-dependent manner, and no significant difference in NF-κB activity was found for doses higher than 6 Gy. Therefore, 6 Gy was used in the following experiments. To elucidate the underlying mechanism, the Huh7/NF-κB-*tk-luc2/rfp* cells were also transfected with the IκBαM vector, to block the activation of NF-κB, and the NF-κB activity was monitored by bioluminescent imaging (BLI). The NF-κB activity was significantly inhibited as shown in Figure 1B, while radiation-induced cytotoxicity was significantly enhanced (Figure 1C). In addition, the IκBαM vector transfection suppressed the expressions of radiation-induced NF-κB downstream proteins such as VEGF, MMP-9, XIAP, Bcl-2, and cyclinD1 (Figure 1D). These results suggest that inhibition of NF-κB activity could augment the radiosensitivity of Huh7/NF-κB-*tk-luc2/rfp* cells through suppressing expressions of radiation-induced NF-κB downstream proteins.

### Sorafenib inhibits Huh7/NF-κB-tk-luc2/rfp, HepG2, and Hep3B cell growths through NF-κB/ERK pathway

**Figure 2:**
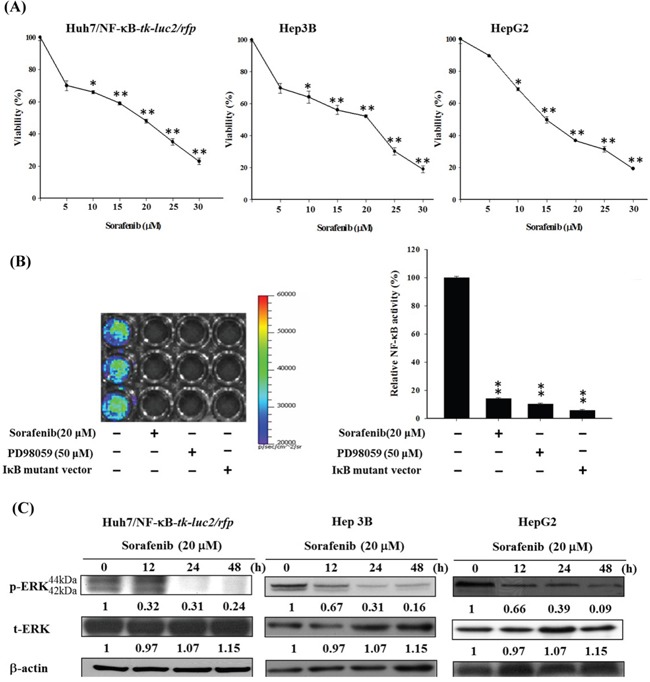
Sorafenib induces cytotoxicity and apoptosis in Huh7/NF-κB-*tk-luc2/rfp*, HepG2, and Hep3B cells *via* downregulating the ERK pathway **A.** Three human HCC cell lines were treated with various concentrations (5–25 μM) of sorafenib for 48 h, and the viabilities were measured byMTT assay. **B.** The luciferase reporter gene assay indicated that sorafenib suppressed the NF-κB activation effectively as PD98059, a MEK inhibitor, and the IκBαM vector. **C.** Western blotting revealed that sorafenib inhibited the expression of phospho-ERK in a time-dependent manner. (**p< 0.01, as compared with that of the control group).

The cytotoxicity of sorafenib in three human HCC cell lines was assessed using MTT assay (Figure 2A). The half maximal inhibitory concentrations (IC_50_)of these cell lines were about 15–20 μM. In Huh7/NF-κB-*tk-luc2/rfp* cells, NF-κB activation was markedly suppressed by both sorafenib and PD98059, a MEK inhibitor (Figure 2B). Figure 2C shows that sorafenib markedly inhibits the expression of p-ERK in a time-dependent manner up to 48 hours, suggests that sorafenib could effectively inhibit NF-κB activity through dephosphorylation of ERK.

### Synergistic effect of sorafenib combined with RT in HCC cells

**Figure 3:**
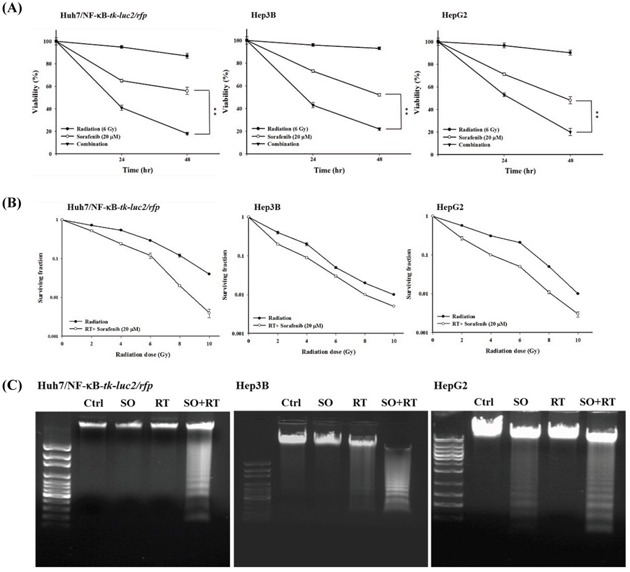
Combination therapy significantly enhances cytotoxicity and apoptosis in all three human HCC cell lines **A.** Three human HCC cell lines were treated with 20 μM sorafenib, 6 Gy irradiation, or the combination therapy for 24 and 48 h. MTT assay was performed to assess the viabilities, and the highest cytotoxicity was observed in cells receiving combination therapy in all three cell lines. **B.** The clonogenic assay was performed to establish the radiation survival curves of cells treated with and without 20 μM sorafenib. The radiation survival curves of three cell lines were left-shifted when the combination therapy was given. **C.** The combination therapy resulted in the most obvious DNA fragmentation among all the treatments. (*p< 0.05, **p< 0.01 as compared with the control group; #*p*< 0.05, ##*p*< 0.01 as compared with the RT group).

The viability and surviving fractions of three HCC cell lines were determined with MTT assay (Figure 3A) and clonogenic assay (Figure 3B), respectively. The cytotoxicity of combination treatment compared with either single treatment was significantly higher in these cells. The combination effects were evaluated according to the formulas proposed by Valeriote and Lin and Carpentier *et al* [34, 35], and were listed in Tables 1–3. Synergistic cytotoxicity was found when various radiation doses (2–10 Gy) combined with 20 μM sorafenib for all three cell lines. Increased DNA fragmentation was also found in the combination treatment compared with that of either single treatment in all three cell lines (Figure 3C).

**Table 1:** The effects of sorafenib combined with radiation on Huh7/NF-κB-*tk-luc2* cells

Radiation (Gy)	SF_R_	SF_R+S_	SF_R_ x SF_S_
2	0.72	0.52 (Synergism)*	0.50
4	0.54	0.24 (Synergism)*	0.38
6	0.29	0.12 (Synergism)*	0.20
8	0.12	0.02 (Synergism)*	0.08
10	0.04	0.004 (Synergism)*	0.03

The surviving fraction of sorafenib is 0.7

* According to formulas suggested by Valeriote and Lin and Carpentier *et al*

Synergism, SF_R+S_< SF_R_ x SF_S_;

Additivity, SF_R+S_ = SF_R_ x SF_S_;

Sub- additivity, SF_R+S_> SF_R_ x SF_S_ with SF_R+S_< SF_R_ and SF_R+S_< SF_S_;

Antagonism, SF_R+S_> SF_R_ and/or SF_R+S_> SF_S_

SF_R_ = Surviving fraction of Huh7/NF-κB-*tk-luc2*, Hep3B and HepG2 cells treated with radiation alone.

SF_S_ = Surviving fraction ofHuh7/NF-κB-*tk-luc2*, Hep3B and HepG2 cells treated with 20 μM sorafenib alone.

SF_R+S_ = Surviving fraction ofHuh7/NF-κB-*tk-luc2*, Hep3B and HepG2 cells treated with radiation plus sorafenib 20 μM.

**Table 2:** The effects of sorafenib combined with radiation on Hep3B cells

Radiation (Gy)	SF_R_	SF_R+S_	SF_R_ x SF_S_
2	0.40	0.20 (Synergism)	0.28
4	0.20	0.09 (Synergism)	0.14
6	0.05	0.03 (Synergism)	0.04
8	0.02	0.01 (Synergism)	0.01
10	0.01	0.005 (Synergism)	0.007

The surviving fraction of sorafenib is 0.7.

**Table 3:** The combined effect on HepG2 cells was assayed, and calculated according to the method of Valeriote and Lin and Carpentier

Radiation (Gy)	SF_R_	SF_R+S_	SF_R_ x SF_S_
2	0.40	0.27 (Synergism)	0.40
4	0.30	0.10 (Synergism)	0.21
6	0.21	0.05 (Synergism)	0.15
8	0.05	0.01 (Synergism)	0.04
10	0.01	0.001 (Synergism)	0.007

The surviving fraction of sorafenib is 0.7.

### Sorafenib inhibits the NF-κB/DNA binding activity and expressions of NF-κB downstream proteins induced by radiation in HCC cells

**Figure 4:**
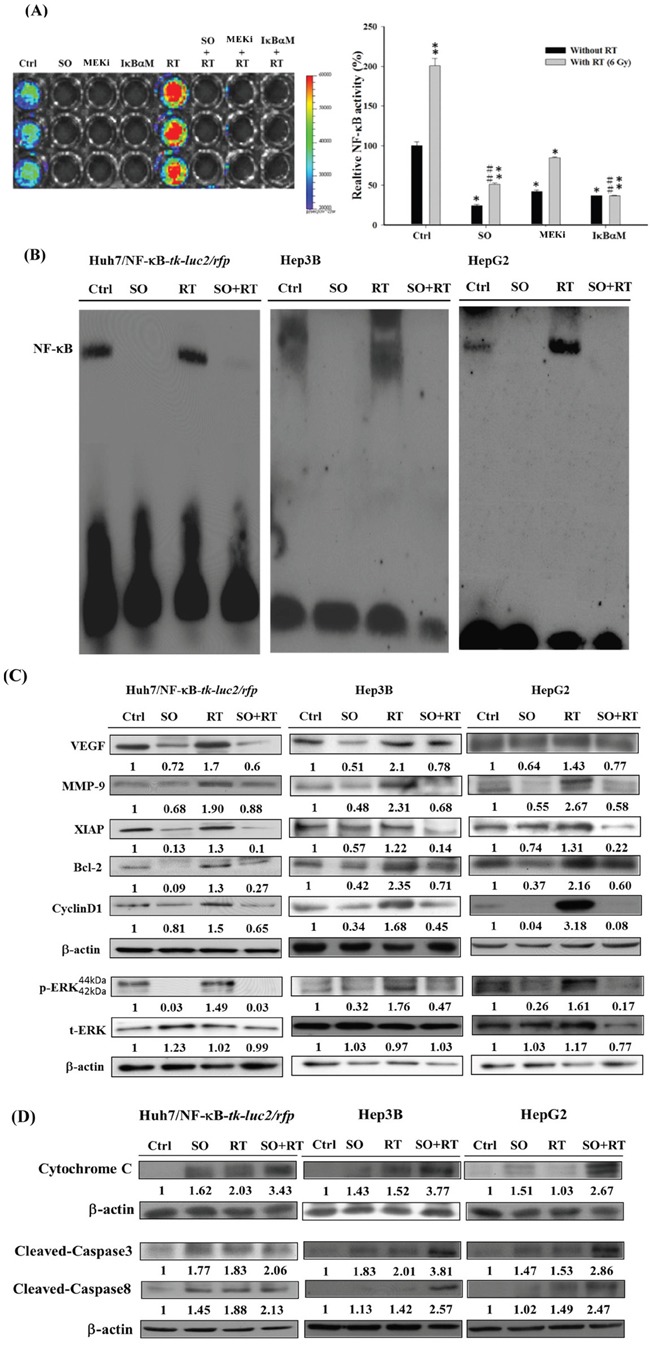
Sorafenib inhibits the radiation-induced activity of NF-κB and its downstream proteins expressions *via* downregulating the ERK pathway **A.** Huh7/NF-κB-*tk-luc2/rfp* cells treated with 20 μM sorafenib, 50 μM PD98059 and transfected with the IκBαM vector were irradiated with or without 6 Gy radiation. Sorafenib, PD98059, and the IκBαM vector significantly suppressed NF-κB activity induced by radiation. **B.** EMSA was utilized to determine the DNA binding activity, which was suppressed by the combination therapy in all three HCC cell lines. **C.** The combination therapy reduced the expressions of NF-κB-regulated proteins induced by radiation. **D.** The combination therapy caused cell death through upregulating the expressions of apoptosis-related proteins. (*p< 0.05, **p< 0.01 as compared with the control group; #p < 0.05, ##p < 0.01 as compared with the RT group).

The NF-κB activity induced by radiation in HCC cells was inhibited effectively by sorafenib, PD98059, and the IκBα mutant vector determined by the luciferase reporter gene assay (Figure 4A). Radiation-induced NF-κB/DNA binding activity could be inhibited by sorafenib as well (Figure 4B). In addition, Western blotting showed that radiation-induced expressions of VEGF, MMP-9, XIAP, Bcl-2, cyclinD1 and p-ERK could be suppressed by sorafenib in all three cell lines (Figure 4C). Moreover, expressions of both mitochondria-dependent and -independent apoptotic proteins, including cleaved caspase-3, cleaved caspase-8, and cytochrome *C* were significantly increased when combined radiation with sorafenib (Figure 4D).

### Pretreatment with sorafenib combined with RT inhibits tumor growth in Huh7/NF-κB-tk-luc2/rfp tumor-bearing mice

**Figure 5:**
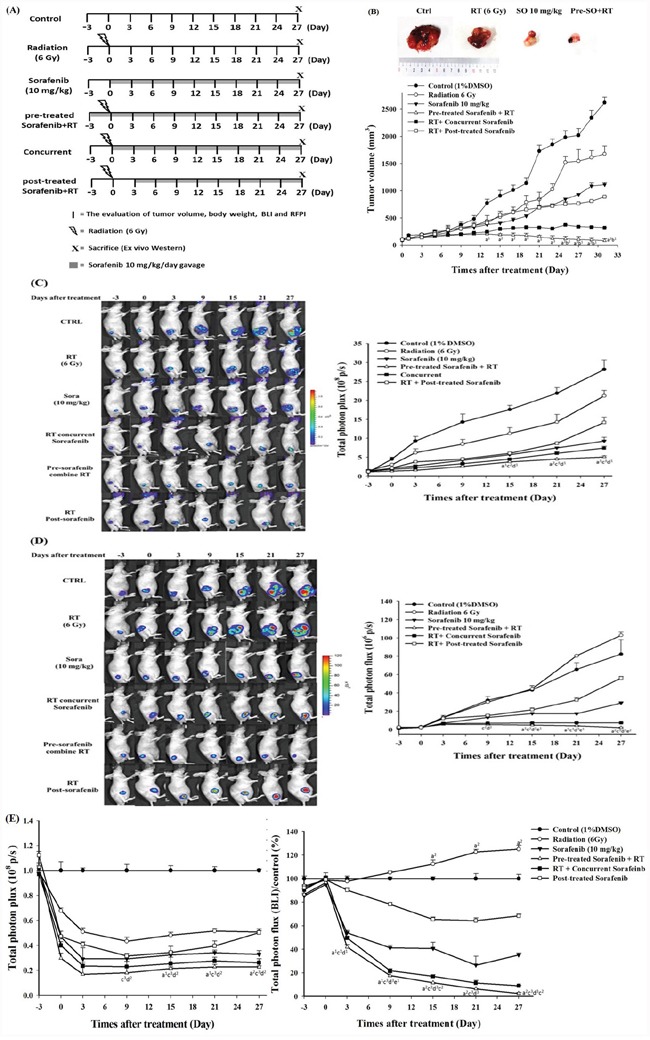
Sorafenib enhances therapeutic efficacy in HCC tumor-bearing mice. **A.** Illustration of various treatment schedule of the *in vivo* experiments **B.** Upper panel: representative tumors harvested from each group on day 27. Lower panel: tumor growth curves determined by caliper measurement. The tumor volume was markedly decreased by the pretreatment of sorafenib plus RT. **C.** Left panel: RFP image reflected the numbers of viable cells in tumors and a substantial decrease in signal intensity was found in the pretreatment of sorafenib plus RT group. Right panel: quantification data of the RFP images. **D.** Left panel: the NF-κB activity of each mouse was monitored with BLI longitudinally. Right panel: quantification results of NF-κB activity detected by BLI. **E.** Left panel: all the quantified RFP signals were normalized to the signal derived from the control group. The RFP signals decreased in all the groups receiving treatments as compared with the control group. Right panel:normalized NF-κB activity signals obtained from all the groups. The signals increased significantly in the RT group as compared with the control group. (a vs. post-treatment, b vs. concurrent, c vs. control, d vs. RT, and e vs. sorafenib ^1^: p < 0.05, ^2^: p < 0.01, and ^3^: p< 0.005).

Radioresistance could be reduced by sorafenib through downregulation of NF-κB/ERK pathway (Figures 3A, 3B, 4A and 4B) suggests that combination treatment might augment the tumor inhibition *in vivo*. However, the administration sequence of sorafenib and RT may affect substantially the treatment outcome *in vivo*. Here three combination schedules were performed to determine the optimal treatment sequence. Mice were randomly divided into six groups as described in the Materials and Methods section: control, sorafenib alone (10 mg/kg/d by gavage), RT alone (single 6 Gy), pretreatment, concurrent, and post-treatment groups. Tumor volumes were measured with a digital caliper three times per week. Both NF-κB activity and *rfp* signal levels of living cells in the tumor were detected by IVIS50 twice a week. Mice were sacrificed on day 27 after treatment to perform the whole-body autoradiography. Tumors obtained from each group were assayed for *ex vivo* Western blotting and EMSA (Figure 5A). Combination treatments showed better tumor growth inhibition as compared with either single treatment. Notably, the best tumor growth inhibition was found in the pretreatment group. The mean tumor volume of the pretreatment group was significantly smaller than that of the post-treatment group and concurrent group from day 13 and day 25, respectively, till the end of the experiment (Figure 5B). The tumor growth of each group was monitored by the red fluorescent protein imaging (RFPI) which represent the numbers of viable cells as shown in Figure 5C. The quantified results showed that there were significant differences discovered when compared the pretreatment group with the control, RT alone, and post-treatment groups. Both the tumor growth curve and the RFPI indicated that the pretreatment resulted in the best tumor inhibition. The *in vivo* NF-κB activity was visualized by the BLI. The total photon fluxes emitted from tumors of the RT alone group were similar to that of the control group, but slightly increased at the later time points. All the sorafenib treated groups including the three combination treatments had lower total photon fluxes as compared with the control and RT groups (Figure 5D). The results imply that radiation-induced NF-κB activity could be repressed by sorafenib treatment. Both the RFPI and BLI results of each group were normalized to the results of the control group and plotted in the Figure 5E. The lowest RFPI and BLI signals were found in the pretreatment and concurrent groups, which had the smallest tumor volumes in the tumor growth curve. The increased toxicity to normal tissue should always be considered when combination treatment is used, and the general toxicity was evaluated by body weight tracking. No significant body weight loss was observed in all the treatment groups as compared with the control group.

### Sorafenib pretreatment combined with RT markedly inhibits the tumor growth via suppressing the expression of NF-κB and increasing the expressions of apoptotic proteins

**Figure 6:**
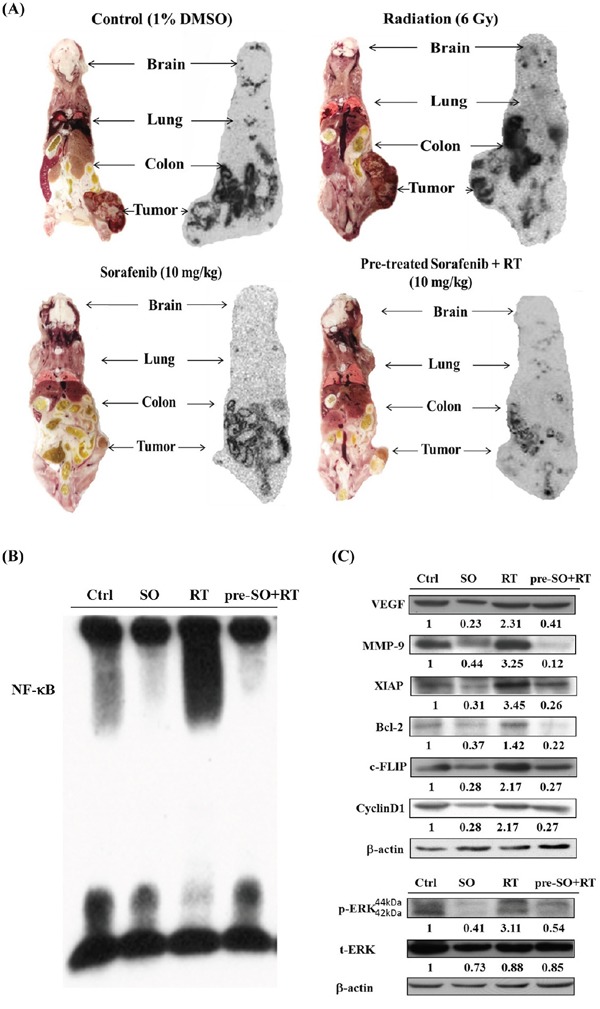
NF-κB activity and its downstream proteins are suppressed by pretreatment of sorafenib plus radiation. **A.** The NF-κB activity was assessed by the expression level of *tk* represented by the uptake of ^131^I-FIAU, which was decreased most significantly in the pretreatment of sorafenib plus RT group **B.** NF-κB/DNA binding activity was evaluated with EMSA, and NF-κB activity was strongly elevated by radiation and suppressed in the pretreatment of sorafenib plus RT group. **C.** The expressions of NF-κB downstream proteins were detected by Western blotting, and substantial decreases were found in the group pretreated with sorafenib plus RT.

Mice were sacrificed on the day 27 post-treatment, and the activity of NF-κB and its downstream proteins within tumors were evaluated. Because the HSV-1 *tk* gene was driven by the NF-κB responsive element as the *luc2* gene, and the uptake of ^131^I-FIAU was determined by autoradiography to reflect the NF-κB activity detected by BLI. As seen in BLI, little uptakes of ^131^I-FIAU were found in the sorafenib and pretreatment groups (Figure 6A). The *ex vivo* EMSA echoed the autoradiography findings. Radiation did induce the NF-κB activity *in vivo*, and pretreatment of sorafenib markedly suppressed the induction (Figure 6B). Furthermore, the pretreatment group exhibited lower expressions of NF-κB downstream proteins as compared with other groups (Figure 6C).

## DISCUSSION

Although RT has been used for treating unresectable HCC for many years, numerous studies have shown that radiation may promote the malignant behavior of cancer cells by activating several signaling pathways that are involved in tumor invasiveness, metastasis, proliferation, and antiapoptosis [36–38]. Moreover, NF-κB has been proposed to play a crucial role in controlling the dynamic balance between radiation-induced apoptosis and resistance [39, 40]. Here the level of NF-κB and expressions of its downstream proteins induced by radiation in Huh7/NF-κB-*tk-luc2/rfp* cells were shown to be suppressed by transfected with the IκBαM vector and resulted in higher radiation cytotoxicity, suggesting that inhibition of NF-κB could enhance the radiosensitivity of Huh7, a human HCC cell line (Figure 1). Hence targeting NF-κB activation may be a novel strategy for HCC treatment. Moreover, the Huh7/NF-κB-*tk-luc2/rfp* stable clone was established for screening the potential drugs, which could suppress the activation of NF-κB. Sorafenib inhibits the proliferation and angiogenesis of tumor cells through targeting Raf, VEGF, PDGF receptor tyrosine kinase signaling and downregulating of Ras/MEK/ERK signaling pathway [41, 42]. Here sorafenib was revealed to inhibit the NF-κB activity and increase the cytotoxicity, and the time-dependent suppression of p-ERK was found by Western blotting in HCC (Figures 2A-2C). In our previous studies, we have shown that sorafenib could function as a NF-κB inhibitor and a potential radiosensitizer [17, 18]. Thus, combination of sorafenib and RT was conducted to examine whether sorafenib could radiosensitize the HCC cells through reducing the radiation-induced NF-κB activity. Enhanced cytotoxicity induced by radiation was observed in cells treated with combination therapy using MTT and clonogenic assays (Figure 3A and 3B). Radiation causes the cell death mainly through apoptosis, which could be evaluated by DNA fragmentation assay. The results show the significant DNA laddering in three HCC cell lines treated with combination therapy (Figure 3C). Increased NF-κB activity induced by radiation *via* modulating the MEK signaling pathway was observed (Figure 4A and 4B). The expressions of NF-κB downstream proteins induced by radiation were also suppressed by combination therapy (Figure 4C). Moreover, both mitochondria-dependent and -independent apoptotic pathways were highly activated in cells treated with combination therapy (Figure 4D).

Combination effect of sorafenib and radiation on tumor growth inhibition has been reported to be more effective with concurrent than post-treatment in HCC *in vitro* but not *in vivo*, suggesting a schedule-dependent effect of the combination of sorafenib and RT on HCC [19, 21]. Accordingly, the optimal sequential course for the combination of RT and sorafenib and the underlying mechanisms need to be clarified. Notably, in this study the pretreatment of sorafenib plus RT demonstrated the most significant tumor growth inhibition due to the suppression of radiation-induced NF-κB and its downstream proteins.

Sorafenib could enhance the cytotoxicity of radiation by increasing intrinsic radiosensitivity and impairing the sublethal DNA damage repair in SMMC-7721 and SK-HEP-1 HCC cell lines, and increased the antitumor effects of radiation in two tumor-bearing mouse models whether concurrent or post-treatment of sorafenib were taken, which suggests that further studies of the optimal sequence are necessary [20]. Here three treatment schedules of combination therapy were performed as depicted in Figure 5A. Mice received combination therapy showed better tumor control than those treated with RT alone indicating that sorafenib could be a radiosensitizer. Notably, the highest tumor growth inhibition was found in the group with pretreatment of sorafenib followed by RT (Figure 5B). This finding was further verified by *in vivo* RFPI and BLI which demonstrated that the sorafenib alone group had lower NF-κB activity compared with those of the control, RT alone and RT followed by post-treatment of sorafenib (Figure 5C). Noteworthy, the levels of NF-κB activity within tumors seemed to be correlated to the treatment schedule of sorafenib combined with RT. Pretreatment of sorafenib followed by RT shows the lowest NF-κB activity in Huh7/NF-κB-*tk-luc2/rfp* tumor-bearing mouse model (Figure 5D). These results suggest that sorafenib sensitizes HCC to radiation is dependent on the sequential order of treatment schedule. Sorafenib exhibits radiosensitization effects through inhibiting both endogenous and radiation-induced NF-κB activity. Hence, sorafenib pretreatment is suggested to enhance the therapeutic efficacy of RT. Though combination treatment results in better tumor control, hepatic toxicity remains to be concerned even when SBRT was used to deliver the radiation dose [22–24]. Based on the monitoring of body weight change, no general toxicity was found in combination treatments in this study. This may be due to the small dosage of sorafenib used here compared with that of the clinical setting. The side-effects were reported when sorafenib was administered with 600 mg daily, but could be reduced using 400 mg daily [23]. Since GI bleeding was observed using combination of sorafenib and RT for large HCC, the patient with small tumor might be benefited using low sorafenib dose (200 mg po bid) and less volume of normal tissue irradiated [24]. Here targeting MEK/ERK/NF-κB pathway by pretreatment of sorafenib to reduce the radioresistance in HCC showed the better tumor inhibition compared with concurrent or post-treatment groups.

**Figure 7:**
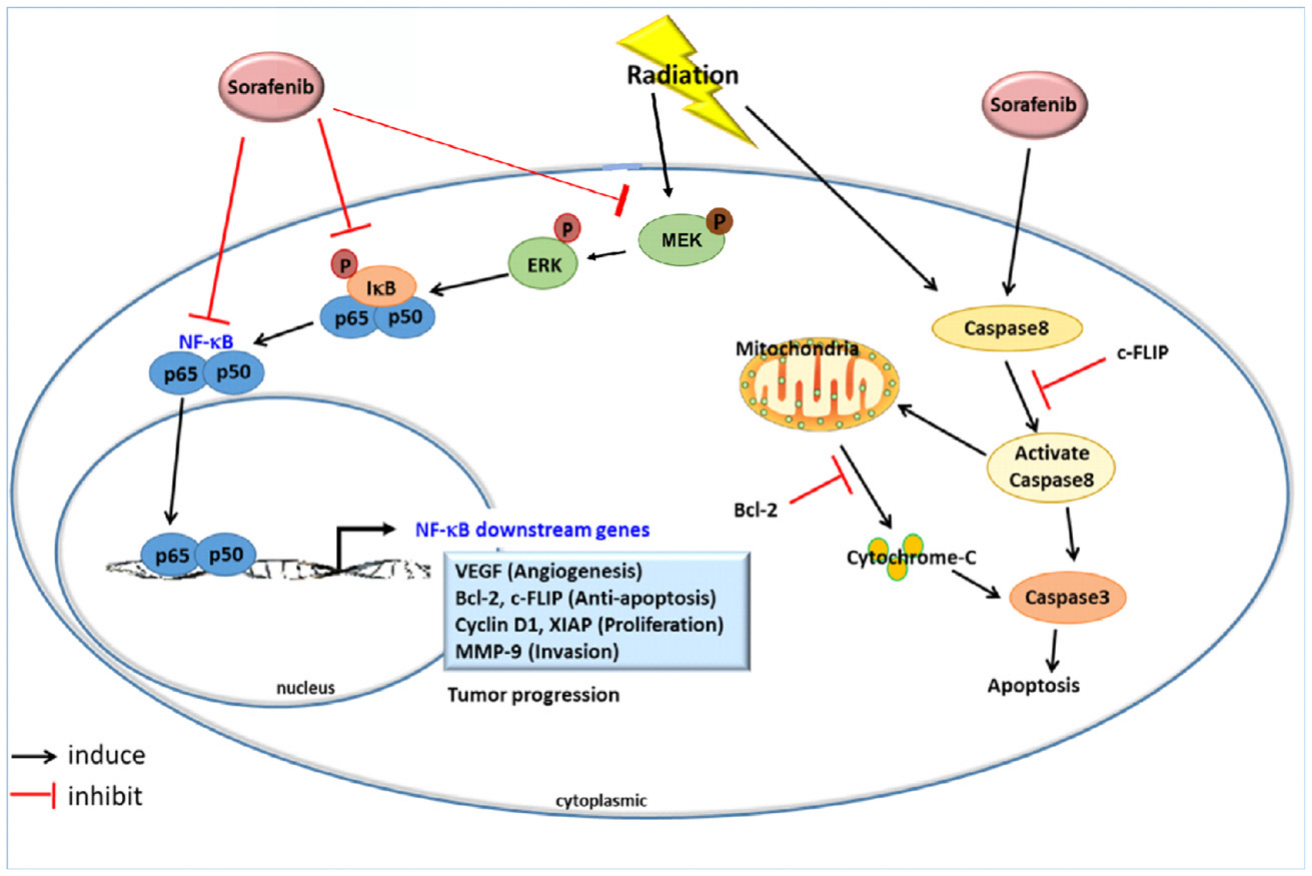
Proposed mechanisms that pretreatment of sorafenib plus RT enhances the therapeutic efficacyin HCC Both sorafenib and RT could trigger cell deaths through apoptotic pathways; however, RT also induces NF-κB activity *via* ERK phosphorylation and results in upregulations of NF-κB downstream proteins. Sorafenib has been proved to inhibit the NF-κB activity induced by RT and avoids the development of radioresistance in HCC in the present study.

Based on our previous findings and the results obtained from this study, the proposed underlying mechanisms of sorafenib pretreament combined with radiation for human HCC are illustrated in Figure 7 [17, 18, 28]. NF-κB plays an important role in controlling the outcomes of cancer treatment since it is a relay molecule in many signaling pathways related to tumor progression, angiogenesis, and metastasis. RT results in cell death through upregulation of apoptotic pathways, but elevates the NF-κB activity *via* ERK pathway in HCC cells. Sorafenib blocks not only the endogenous NF-κB activity but that induced by RT. Moreover, sorafenib causes the apoptosis through both mitochondria-dependent and - independent pathways. Accordingly, pretreatment of sorafenib plus RT could provide the better tumor growth inhibition than any single or combination treatments. In addition, the NF-κB imaging platform provides a real-time method for tracing NF-κB activity *in vitro* and *in vivo,* and may be beneficial for determining the optimal treatment schedule in tumor-bearing animal model.

## MATERIALS AND METHODS

### Cell culture

Human hepatocellular carcinoma cell line Huh7 was provided by Dr. Chia-Hsien Cheng at the Department of Radiation Oncology, National Taiwan University Hospital, Taipei, Taiwan. Hep3B and HepG2 cell lines were obtained from Dr. Shu-Ling Fu at the Institute of Traditional Medicine, National Yang-Ming University. All cell lines were cultured in Dulbecco's minimum essential medium (DMEM) (Hyclone, Logan, UT, USA) containing 10% fetal bovine serum (Hyclone) and 1% penicillin/streptomycin (Gibco^®^, Grand Island, NY, USA). Except for 500 μg/mL G418 (Calbiochem^®^, La Jolla, CA, USA) was also added to the Huh7/NF-κB-*tk-luc2/rfp* stable clone established previously [33]. All cells were maintained at 37°C in an humidified incubator with 5% CO_2_.

### Plasmid transfection

2×10^6^ Huh7/NF-κB-*tk-luc2/rfp* cells seeded into 10 cm were transfected with the IκBα mutant vector (p-IκBαM; Clontech) using the protocol provided by jetPEI^TM^ reagent (Polyplus Transfection, NY, USA) for NF-κB inhibition. Briefly, 8 μg DNA and 16 μL jetPEI diluted with 500 μL of 150 mM NaCl were mixed and incubated at room temperature for 20 min prior to transfection. BLI and electrophoretic mobility shift assays (EMSA) were performed to confirm the inhibitory effect of the IκBα mutant vector on NF-κB activity.

### Irradiation

Three exponentially growing HCC cell lines were exposed to various doses of radiation (0–10 Gy) by using a RS 2000 X-ray Biological Irradiator (RS 2000; Rad Source Technologies, Suwanee, GA, USA). The dose rate was 1.03 Gy/min, and the source-to-bolus distance was 80 cm. For *in vivo* studies, tumor-bearing mice were immobilized in lead-shielded jigs with only the right hind leg exposed to X-ray.

### Preparation of sorafenib

Sorafenib was extracted from the Nexavar® tablets (Bayer Healthcare Co., St. Louis, USA) and prepared as described in the previous study [17]. For *in vitro* experiments, sorafenib was first dissolved in 100% dimethyl sulfoxide (DMSO; Sigma, St Louis, MO, USA) and diluted with DMEM with the final DMSO concentration of 0.1%. For *in vivo* study, each mouse was administered with10 mg/kg/d of sorafenib dissolved in double-distilled water by gavage.

### Animals

Eight-week-old male BALB/c nude mice were purchased from the National Laboratory Animal Center, Taiwan. For tumor implantation, 5 × 10^6^ Huh7/NF-κB-*tk-luc2/rfp* cells were inoculated into the right hind legs of the mice. Tumor volume was measured with a digital caliper and calculated with the equation: tumor volume = 0.523 × length × width^2^. When the mean tumor volume reached 70 mm^3^, the mice were divided into the following six groups based on the received treatment: control group, sorafenib alone group (10 mg/kg/d), RT alone group (6 Gy), pretreatment group (sorafenib 3 d before RT), concurrent group (RT plus concurrent sorafenib), and post-treatment group (sorafenib 3 d after RT). All animal protocols were in compliance with the guidelines of the Animal Care and Use Committee at National Yang-Ming University and the 3Rs principle for animal experimentation with animal protocol number: 1001238.

### Clonogenic assay

All three HCC cell lines were pretreated with or without 20 μM sorafenib for 4 h and then treated with various doses of radiation (0–10 Gy) as previously described. The cells were subsequently trypsinized and reseeded in increasing order according to the receiving radiation doses and incubated at 37°C for 14–15 days. Colonies were fixed with methanol and acetic acid and stained with 2% crystal violet, and colonies containing ≥50 cells were counted. The surviving fraction was calculated using the following formula: surviving fraction = colonies counted/number of cells seeded × plating efficiency.

### 3-(4,5-dimethylthiazol-2-yl)-2,5-diphenyltetrazolium bromide assay (MTT assay)

All three HCC cell lines were seeded in 96-well plates (2 × 10^4^ cells/well) a day before treatments. The cells were treated with 0–25 μM sorafenib, 6 Gy irradiation, or a combination of these two treatments, and the cells were incubated for another 48 h. After the cells were washed with fresh medium, 100 μL medium containing 0.5 mg/mL MTT was added to each well. After 2 h of incubation at 37°C, 100 μL of DMSO was added into the wells to dissolve the MTT formazan. Finally, the absorbance of each well was determined using an ELISA reader (Power Wave X340; Bio-Tek Instrument Inc., Winooski, VT, USA) with an excitation wavelength of 570 nm.

### Luciferase reporter gene assay - NF-κB activity

Huh7/NF-κB-*tk-luc2/rfp* cells were seeded into a 96-well plate (3 × 10^4^/well) a day before treated with 50 μM PD98059, 20 μM sorafenib, 0–10 Gy of radiation, or sorafenib and radiation followed by incubation for 48 h. Subsequently, 100 μL of 500 μM *D*-luciferin (Gold Bio Technology, St. Louis, MO, USA) was added into each well. Signal emitted from each well was acquired for 1 min using IVIS50 Imaging System (Xenogen, Hopkinton, MA, USA) and quantified into photons per second by using Living Image software (Xenogen). Results obtained from each group were compared with the DMSO-treated controls to calculate the relative NF-κB activity.

### Electrophoretic mobility shift assay (EMSA)

Cells were treated with 20 μM sorafenib, 6 Gy of irradiation, or the combination treatment and then incubated for 48 h. Nuclear protein was extracted followed the procedures provided by the Nuclear Extraction Kit (Millipore, Billerica, MA, USA). The following DNA sequences were synthesized for EMSA analysis: sense; AGTTGAGGGGACTTTCCCAGGC, and antisense; GCCTGGGAAAGTCCCCTCAAC. The NF-κB/DNA binding activity was measured using the LightShift Chemiluminescent EMSA kit (Pierce), and all steps in the protocol were followed.

### Western blotting

HCC cells were treated in the same way as described in the EMSA assay. 40 μg of total protein was separated using 10-15% SDS-PAGE, then transferred to polyvinylidene difluoride membrane (Millipore), blocked with 5% nonfat milk in TBS-Tween 20 buffer (0.1% Tween 20) for 1 h at room temperature. The membrane was incubated with primary antibodies against proteins of interest including VEGF, Bcl-2, cyclin D1, MMP-9, caspase-3, phospho-ERK (Millipore), and XIAP (Abcam, Cambridge, UK) overnight at 4°C. Membrane was further incubated with horseradish-peroxidase-conjugated secondary antibodies for 30 min at room temperature, and was detected using enhanced chemiluminescence system (Millipore). Image J software (National Institutes of Health, Bethesda, MD, USA) was used for quantitative analysis. β-actin was used as an internal control, and the protein expression levels of the treated groups were compared with those of the control group.

### DNA fragmentation

All three HCC cell lines were seeded into 6-well plate24 h before treatments. The cells were treated with 20 μM sorafenib, 6 Gy irradiation, or a combination treatment, and incubated for another 48 h. The DNA was then isolated and assayed using the DNA purification kit (Axygen, Tewksbury, MA, USA). The DNA was analyzed with 0.7% DNA agarose gel electrophoresis. Finally, DNA fragmentation was observed using the Wealtec Dolphin-View 1147001USB gel imaging system (Wealtec Bioscience, NV, USA).

### Whole-body autoradiography

Mice were injected intravenously with 3.7×10^6^ Bq/0.2 mL ^131^I-1-(2-deoxy-2-fluoro-1-D-arabinofuranosyl)-5-iodouracil (^131^I-FIAU) on day 27 post-treatment and sacrificed 24h after injection. For whole-body autoradiography, mice were frontally sectioned with 30 μm thicknesses at −20 °C using the cryostat microtome (Bright Instruments, Huntingdon, Cambridgeshires, UK). Sections were placed on an imaging plate (BAS-SR2040, Fuji Photo Film, Tokyo, Kanto, Japan) in a cassette to generate phosphor images. The phosphor images were acquired by scanning the imaging plates with the FLA5000 reader after exposure. The reading parameters of the reader were shown as follows: a resolution of 10 μm, a gradation of 16 bits, laser light wavelength of 635 nm, and photomultiplier tube voltage of 800 V.

### Statistical analysis

All data are shown as means ± standard errors. Student's *t* test was used for comparison between two groups. Kaplan-Meier plotting was used for the survival analysis, and was compared using the log-rank test. Differences between the means were considered significant if *P < 0.05 and **P < 0.01.
